# A Thirty Million Year-Old Inherited Heteroplasmy

**DOI:** 10.1371/journal.pone.0002938

**Published:** 2008-08-13

**Authors:** Vincent Doublet, Catherine Souty-Grosset, Didier Bouchon, Richard Cordaux, Isabelle Marcadé

**Affiliations:** Université de Poitiers, Laboratoire Ecologie, Evolution, Symbiose, UMR CNRS 6556, Poitiers, France; National Institute on Aging, United States of America

## Abstract

Due to essentially maternal inheritance and a bottleneck effect during early oogenesis, newly arising mitochondrial DNA (mtDNA) mutations segregate rapidly in metazoan female germlines. Consequently, heteroplasmy (i.e. the mixture of mtDNA genotypes within an organism) is generally resolved to homoplasmy within a few generations. Here, we report an exceptional transpecific heteroplasmy (predicting an alanine/valine alloacceptor tRNA change) that has been stably inherited in oniscid crustaceans for at least thirty million years. Our results suggest that this heteroplasmy is stably transmitted across generations because it occurs within mitochondria and therefore escapes the mtDNA bottleneck that usually erases heteroplasmy. Consistently, at least two oniscid species possess an atypical trimeric mitochondrial genome, which provides an adequate substrate for the emergence of a constitutive intra-mitochondrial heteroplasmy. Persistence of a mitochondrial polymorphism on such a deep evolutionary timescale suggests that balancing selection may be shaping mitochondrial sequence evolution in oniscid crustaceans.

## Introduction

Metazoan mitochondrial genomes evolve rapidly, thus creating extensive sequence variation among individuals within species. While mitochondrial DNA (mtDNA) mutations are widely used in evolutionary studies, they also play a central role in apoptosis, disease and aging [1], [2]. Due to essentially maternal inheritance and an mtDNA bottleneck during early oogenesis, most individuals are homoplasmic for a single mtDNA genome (i.e. they carry a single mtDNA genotype) [2]–[5]. This indicates that newly arising mtDNA mutations segregate rapidly in the female germline. Consistently, evidence from cattle, mice and humans indicates that heteroplasmy (i.e. the mixture of mtDNA genotypes within an organism) is generally resolved to homoplasmy within a few generations [2]–[6]. Therefore, heteroplasmy is considered as a transitional and short-lived state of metazoan mitochondrial evolution. In sharp contrast, we report an unusual transpecific heteroplasmy that has been stably inherited in oniscids (terrestrial isopod crustaceans) for at least 30 million years.

## Results and Discussion

**Figure 1:**
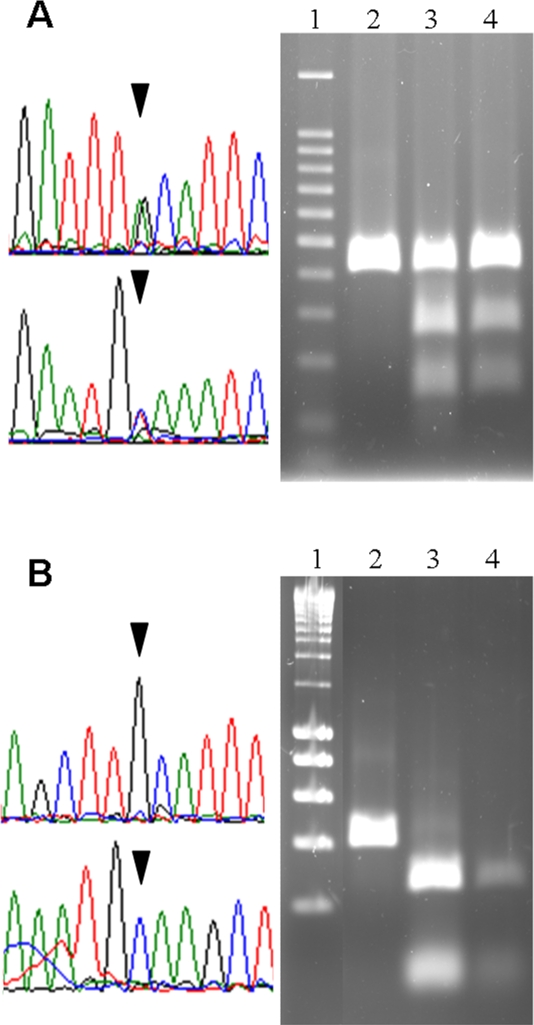
Assessment of character state at nucleotide position 11973 relative to the *Armadillidium vulgare* mitochondrial genome sequence. (A) Presence of the 11973G/A heteroplasmy in mitochondrial DNA extracts of *A. vulgare* individuals. Direct sequencing of a heteroplasmic female sample (left) with forward (top) and reverse (bottom) primers. Partial trace files are shown, with the 11973G/A heteroplasmy highlighted with black triangles. HpyCH4V digestion profiles (right). Lanes: (1) 100 bp molecular ladder (BenchTop, Promega), (2) undigested control amplicon, (3) and (4) digested amplicons of two heteroplasmic female samples. (B) Presence of the 11973G homoplasmic state in *Chaetophiloscia elongata* individuals. Direct sequencing of a homoplasmic individual (left) with forward (top) and reverse (bottom) primers. Partial trace files are shown, with the 11973G homoplasmy highlighted with black triangles. HpyCH4V digestion profiles showing complete digestion of the PCR products (right). Lanes: (1) 200 bp molecular ladder (SmartLadder, Eurogentec), (2) undigested control amplicon, (3) and (4) digested amplicons of two homoplasmic samples.

We have previously identified by direct DNA sequencing a putative G/A heteroplasmic point mutation at nucleotide position 11973 relative to the mitochondrial genome sequence of the oniscid *Armadillidium vulgare*; this polymorphism occurs in a tRNA anticodon and predicts an alanine/valine alloacceptor tRNA change [7]. Interestingly, the 11973G/A heteroplasmy was found in all six individuals tested (five females and one male from various geographic origins) [7]. To independently confirm these results, we designed a PCR-RFLP assay (Fig. 1) and verified the 11973G/A heteroplasmy in these samples. PCR-RFLP analysis of three additional *A. vulgare* individuals (two females and one male) indicated that they all shared the 11973G/A heteroplasmy (data not shown).

To test whether the observed polymorphism is indeed of mitochondrial origin rather than derived from a nuclear mitochondrial pseudogene, we isolated mtDNA of four *A. vulgare* female samples using an alkaline lysis protocol [8]. Specific primers F1 (5′-ACCATCTATGAAGGTTACTGTC) and R1 (5′-ACCAACAGATTCCATACCCAAG) targeting the nuclear actin gene were used in a PCR assay to confirm the absence of nuclear DNA in the samples. We found the 11973G/A heteroplasmy to be present in all four individuals, thus confirming its mitochondrial origin (Fig. 1A).

Presence of the 11973G/A heteroplasmy in all *A. vulgare* individuals analyzed, regardless of their geographic origin and sex, raises the question of its evolutionary origin. To address this issue, we screened 20 isopod crustacean species (from 13 different families) for the presence of the 11973G/A heteroplasmy by direct DNA sequencing and PCR-RFLP. We detected the 11973G/A heteroplasmy in 16 species comprising 10 divergent families of the crustacean suborder Oniscidea [9] (Table 1). These results indicated that the heteroplasmy originally identified in *A. vulgare* actually is a transpecific polymorphism with a very deep evolutionary origin. The fossil record indeed suggests that oniscids arose at least ∼100 million years ago [10] and extent oniscid families are definitely attested by at least ∼34 million years ago [11]. Thus, fossil evidence conservatively suggests that the 11973G/A heteroplasmy may have been present in oniscids for at least ∼30 million years.

**Table 1:** Distribution of the 11973G/A mitochondrial heteroplasmy in 20 isopod crustacean species.

SUBORDER, Family	Species	Origin	N^a^	11973^b^	Assay^c^	PCR primers^d^
FLABELLIFERA						
Sphaeromatidae	*Dynamene bidentata*	France	2	G	F, D	VF2/VR2
ONISCIDEA						
Ligiidae	*Ligia oceanica*	France	3	G	F, R, D	VF1/1737ND
Philosciidae	*Chaetophiloscia elongata*	France	2	G	F, R, D	VF1/1737ND
	*Philoscia muscorum*	France	2	G	F, R, D	VF1/1737ND
Agnaridae	*Hemilepistus reaumuri*	Tunisia	3	G/A	F, R, D	VF2/VR2
Armadillidae	*Armadillo officinalis*	Turkey	2	G/A	F, R	VF2/VR2
	*Cubaris murina*	Guadeloupe	2	G/A	F, R, D	VF2/VR2
Armadillidiidae	*Armadillidium assimile*	France	3	G/A	F, R, D	2138ND/1737ND
	*Armadillidium depressum*	France	3	G/A	F, R, D	2138ND/1737ND
	*Armadillidium nasatum*	France	2	G/A	F, R, D	VF1/1737ND
	*Armadillidium vulgare*	Brazil, France, Greece, Tunisia	26	G/A	F, R, D	2138ND/1737ND
Balloniscidae	*Balloniscus sellowii*	Brazil	1	G/A	F, R, D	VF2/VR2
Cylisticidae	*Cylisticus convexus*	France	3	G/A	F, R, D	VF2/VR2
Platyarthridae	*Platyarthrus hoffmannseggii*	France	1	G/A	F, R	VF2/VR2
	*Platyarthrus caudatus*	Italy	1	G/A	F, R	VF2/VR2
Porcellionidae	*Porcellio gallicus*	France	3	G/A	F, R, D	VF2/VR2
	*Porcellio spinicornis*	France	2	G/A	F, R, D	VF2/VR2
Trachelipodidae	*Trachelipus rathkii*	France	2	G/A	F, R, D	VF1/1737ND
Trichoniscidae	*Trichoniscus pusillus pusillus*	France	1	G/A	F, R	VF2/VR2
Tylidae	*Helleria breviconis*	France	2	G/A	R, D	VF2/VR2

a Number of individuals tested.

b Indicates whether all individuals within species were homoplasmic for guanine (G) or heteroplasmic for guanine and adenine (G/A) at mitochondrial genome position 11973.

c Assay used to ascertain 11973 heteroplasmic status: direct sequencing with forward (F) or reverse (R) primer, and HpyCH4V digestion (D).

d Primer pairs used to amplify the 11973 mitochondrial genome region by PCR: VF1 (5′-CCCGTTTGAGTGTGGGTTTGA), VF2 (5′- TGGTTTTTGATGTTGAGATT), 2138ND (5′-TCCTAGGGATTGGCCATTTA), VR2 (5′- CGCTTACGTTACGATAAACT) and 1737ND (5′- TATTTGGGTGCGAGGAACTC).

**Figure 2:**
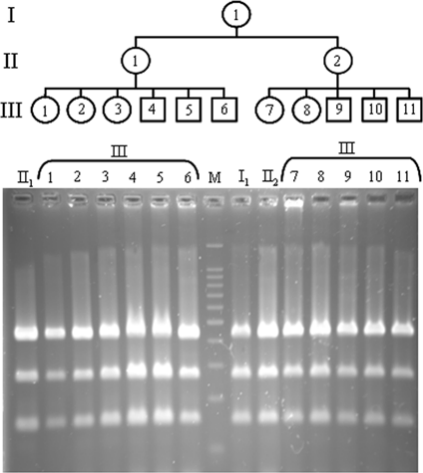
Stable inheritance of the 11973G/A heteroplasmy across three generations (I–III) of an *Armadillidium vulgare* maternal lineage (top). HpyCH4V digestion profiles showing that all individuals carry the 119973G/A heteroplasmy (bottom). M: 100 bp molecular ladder (BenchTop, Promega).

Sharing of a heteroplasmy in such a wide range of species suggests that it may be stably inherited by oniscids. To gain insight into this question, we investigated transmission of the 11973G/A heteroplasmy across three generations of an *A. vulgare* maternal lineage (Fig. 2). The heteroplasmy was found in all individuals of the pedigree (8 females and 6 males), thus demonstrating 100% transmission to both males and females across three generations. Constitutive heteroplasmy has previously been reported in male bivalves [12], [13]. In these species, male and female germlines are homoplasmic for different mtDNA genotypes, each of which is uniparentally transmitted [12], [13]. As a result, females inherit mtDNA following usual strict maternal inheritance and males inherit mtDNA from both parents and, therefore, are heteroplasmic (except in the germline). In sharp contrast with the doubly uniparental mode of mtDNA inheritance of bivalves, both male and female *A. vulgare* gonadic tissues are heteroplasmic (data not shown). Thus, unlike the bivalve system, paternal mtDNA transmission does not need to be invoked in *A. vulgare*. This view is further supported by the fact that we found the 11973G/A heteroplasmy in the parthenogenetic oniscid *Trichoniscus pusillus pusillus* (i.e. females produce eggs that develop without male fertilization) [14]. Therefore, classical strict maternal inheritance of mtDNA in principle can account for the presence of the 11973G/A heteroplasmy in both *A. vulgare* males and females.

How, then, does the 11973G/A heteroplasmy persist in oniscids on the long term? Because of mtDNA bottlenecks during oogenesis, persistence of two distinct populations of mtDNA genotypes within all individuals is very unlikely, unless the homoplasmic state at nucleotide position 11973 is lethal in oniscid species. Yet, mtDNA bottlenecks would predict lethal homoplasmic eggs to be frequently produced, which would seem to induce unbearable reproduction costs to females. Alternatively, stable transmission across generations can be achieved if oniscid mitochondria are constitutively heteroplasmic.

**Figure 3:**
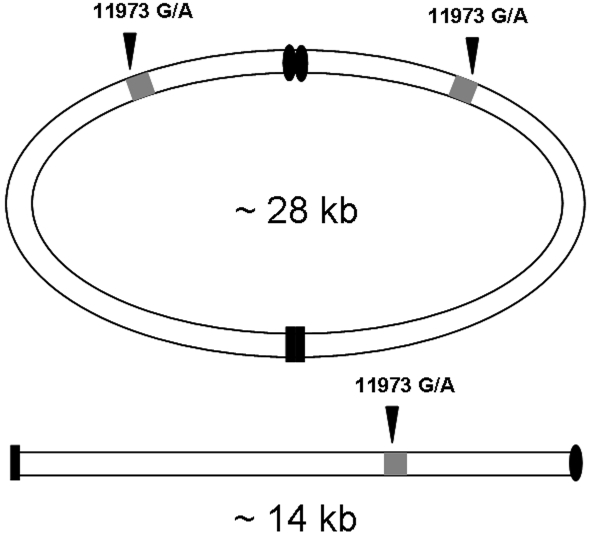
Schematic representation of the atypical structure of *Armadillidium vulgare* mitochondrial genome, highlighting the relative position of the 11973G/A heteroplasmy. The actual replication mechanism of this mitochondrial genome currently is unknown.

Consistently, at least two terrestrial isopod species (*A. vulgare* and *Porcellionides pruinosus*) possess an atypical mitochondrial genome formed by a circular ∼28-kb dimer consisting of two ∼14-kb monomers fused in opposite polarities and a linear ∼14-kb monomer [15], [16] (Fig 3). The circular and linear molecules may not be at equilibrium and the circular state might be generated from the linear state [15]. Such trimeric structure could provide an adequate substrate for the emergence of a constitutive intra-mitochondrial heteroplasmy. Strikingly, the three monomer units of the *A. vulgare* mitochondrial genome are identical at the nucleotide level, except for the 11973G/A heteroplasmy [7]. It is thought that the extreme sequence homogeneity among monomers is maintained by concerted evolution or it may be linked to the replication mechanism of this atypical mitochondrial genome [7]. If so, maintaining a heteroplasmic state for ∼30 million years despite evolutionary forces otherwise tending to homogenize monomer sequences necessarily requires a selective process.

**Figure 4:**
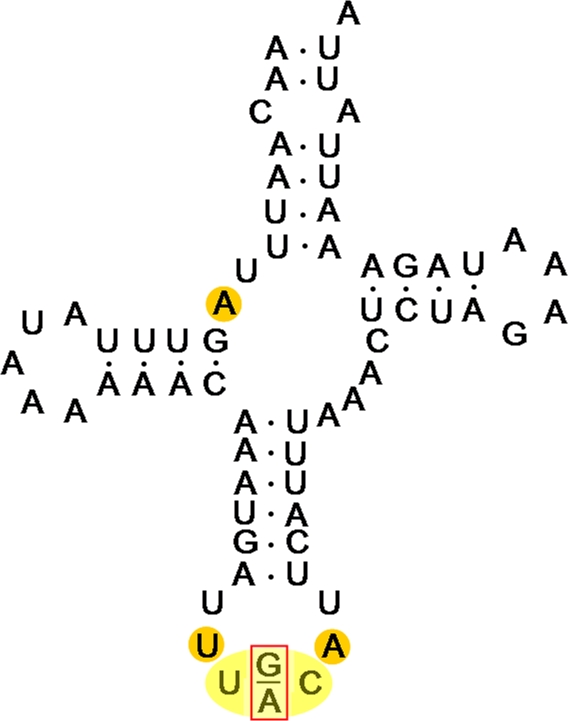
Secondary structure of the dual tRNA^Ala/Val^ from the *Armadillidium vulgare* mitochondrial genome. The 11973G/A heteroplasmy is highlighted in the red rectangle, in the anticodon shown in yellow. Orange circles denote conserved nucleotides for mitochondrial tRNAs [20].

Interestingly, the 11973G/A heteroplasmy occurs in a tRNA anticodon specifying tRNA^Ala^ and tRNA^Val^ [7]. This 65 bp-long tRNA exhibits a typical cloverleaf secondary structure (Fig. 4), as predicted by the tRNAScan-SE program [17], suggestive of biological functionality. In addition, an analysis of the *A. vulgare* mitochondrial genetic code using the GenDecoder program [18] reveals that *A. vulgare* possesses the standard invertebrate mitochondrial genetic code, confirming the use of codons GCA for alanine (used 118 times) and GTA for valine (used 51 times). Thus, tRNA^Ala^ and tRNA^Val^ are both necessary for *A. vulgare* mitochondria to perform proper mRNA translation. Because the tRNA^Ala^ and tRNA^Val^ are apparently encoded nowhere else in the *A. vulgare* mitochondrial genome [7], the tRNA locus exhibiting the 11973G/A heteroplasmy is the sole endogenous source of both tRNA^Ala^ and tRNA^Val^ for *A. vulgare* mitochondria. Since lack of either tRNA could have dramatic consequences on mitochondrial metabolism, the 11973G/A heteroplasmy may have been maintained by a process of balancing selection for the past ∼30 million years of oniscid evolution.

## Materials and Methods

Total DNA was extracted from gonads, fat body and nerve tissues of each individual as previously described [19]. PCR reactions were performed using GoTaq polymerase (Promega), primer combinations shown in Table 1 and the following program: 95°C for 3 min, 35 cycles of 95°C for 30 sec, 51∼54°C for 50 sec, 72°C for 1 min 30, and 72°C for 5 min. Purified PCR products were directly sequenced with the BigDye Terminator kit (Applied Biosystems) and analyzed on an ABI Prism 3130 Genetic Analyzer.

Endonuclease digestion of PCR products were performed with HpyCH4V (New England BioLabs). This enzyme recognizes a TGCA restriction motif (the site corresponding to the 11973 mitochondrial polymorphism is underlined). Digestions were performed with 3.5 units of enzyme (i.e. in excess to avoid partial digestions) at 37°C for 1 h. Digested products were separated on 2% agarose gels in TAE buffer for 1 h 30 at 35 V. Gels were stained with ethidium bromide and examined under UV light. HpyCH4V *in silico* digestion of *A. vulgare* mitochondrial genome [7] predicted a single undigested band (399 bp) in 11973A individuals and two digested bands (250 bp and 149 bp) in 11973G individuals. 11973G/A heteroplasmic individuals exhibited the three bands.
